# Dual‐branch feature fusion S3D V‐Net network for lung nodules segmentation

**DOI:** 10.1002/acm2.14331

**Published:** 2024-03-13

**Authors:** Xiaoru Xu, Lingyan Du, Dongsheng Yin

**Affiliations:** ^1^ School of Automation and Information Engineering Sichuan University of Science and Engineering Zigong People's Republic of China; ^2^ Artificial Intelligence Key Laboratory of Sichuan Province, Sichuan University of Science & Engineering Zigong People's Republic of China

**Keywords:** feature fusion, LUNA16, pulmonary nodule segmentation, Separable 3D, V‐Net

## Abstract

**Background:**

Accurate segmentation of lung nodules can help doctors get more accurate results and protocols in early lung cancer diagnosis and treatment planning, so that patients can be better detected and treated at an early stage, and the mortality rate of lung cancer can be reduced.

**Purpose:**

Currently, the improvement of lung nodule segmentation accuracy has been limited by his heterogeneous performance in the lungs, the imbalance between segmentation targets and background pixels, and other factors. We propose a new 2.5D lung nodule segmentation network model for lung nodule segmentation. This network model can well improve the extraction of edge information of lung nodules, and fuses intra‐slice and inter‐slice features, which makes good use of the three‐dimensional structural information of lung nodules and can more effectively improve the accuracy of lung nodule segmentation.

**Methods:**

Our approach is based on a typical encoding‐decoding network structure for improvement. The improved model captures the features of multiple nodules in both 3‐D and 2‐D CT images, complements the information of the segmentation target's features and enhances the texture features at the edges of the pulmonary nodules through the dual‐branch feature fusion module (DFFM) and the reverse attention context module (RACM), and employs central pooling instead of the maximal pooling operation, which is used to preserve the features around the target and to eliminate the edge‐irrelevant features, to further improve the performance of the segmentation of the pulmonary nodules.

**Results:**

We evaluated this method on a wide range of 1186 nodules from the LUNA16 dataset, and averaging the results of ten cross‐validated, the proposed method achieved the mean dice similarity coefficient (mDSC) of 84.57%, the mean overlapping error (mOE) of 18.73% and average processing of a case is about 2.07 s. Moreover, our results were compared with inter‐radiologist agreement on the LUNA16 dataset, and the average difference was 0.74%.

**Conclusion:**

The experimental results show that our method improves the accuracy of pulmonary nodules segmentation and also takes less time than more 3‐D segmentation methods in terms of time.

## 1 INTRODUCTION

**FIGURE 1:**
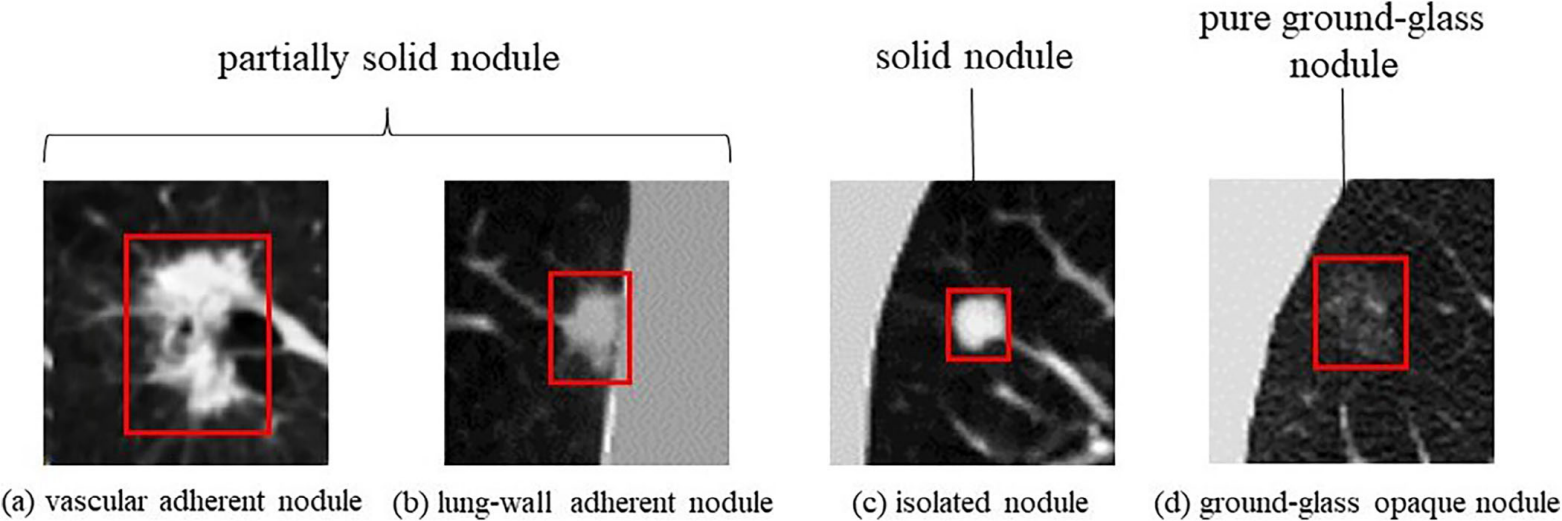
The four types of lung nodules.

Cancer has long been an urgent public health problem. According to the American Cancer Society's statistics on cancer rates and deaths for 2023, lung cancer is the most fatal form of cancer, approximately 350 people die each day from lung cancer.^1^ Currently, low‐dose computed tomography (LDCT) of the chest is the most commonly used lung cancer screening method, which has resulted in a significant increase in the detection rate of pulmonary nodules, and a relative reduction in lung cancer mortality of 20% per year through the diagnosis and treatment of LDCT‐detected pulmonary nodules.^2^ Pulmonary nodules generally appear as rounded, slightly dense, solid, closed shadows between 3 and 30 mm in diameter on CT.^3^ Clinically, the lesions can be classified into three categories according to their size: tiny nodules with a diameter of less than 5 mm, small nodules with a diameter of more than 5 mm and less than 10 mm, and nodules with a diameter of more than 10 mm and less than 30 mm.^4^ However, pulmonary nodules are diverse, random, and non‐standardized in clinical management, and can be classified as solid, pure ground‐glass, and partially solid nodules based on morphological changes. They are also classified as vascular adherent nodules, lung‐wall adherent nodules, isolated nodules, and ground‐glass opaque(GGO) nodules based on location.^5^ Images of the four types of lung nodules are shown in Figure 1.

Segmentation of lung nodules plays an important role in auxiliary diagnosis, treatment planning, and treatment monitoring of lung cancer. Through rapid and accurate segmentation of lung nodules, it can provide an objective size basis for monitoring nodule growth, improve the early diagnosis rate and treatment effect of lung cancer, and enhance better medical services for patients.^6^ Currently, there are six more common and widely used methods for segmenting lung nodules, threshold‐based segmentation method,^7^ region‐growth‐based segmentation method,^8^ edge‐detection‐based segmentation method,^9^ graph‐cut‐based segmentation method,^10^ machine‐learning‐based segmentation method,^11^ and deep‐learning‐based segmentation method.^12^ Among them, deep learning‐based segmentation methods are currently the most advanced lung nodule segmentation methods,^13^, ^14^, ^15^, ^16^ which can achieve automatic and accurate nodule segmentation by learning the features of images through deep neural network models (e.g., U‐Net,^17^ V‐Net,^18^ SegNet,^19^ Mask R‐CNN,^20^ etc.). Deep learning based network models for lung nodule segmentation can be classified into three categories based on the dimensionality of the input data, 2D segmentation models, 3D segmentation models, and 2.5D segmentation models. In 2D network segmentation models, U‐Net and its improved network models are commonly used for segmentation. For example, Ali et al.^21^ proposed two improved U‐Net architectures based on enhanced sensory wilds, which can yield better segmentation results. Dutande et al.^22^ proposed an improved residual squeezing excitation module based on U‐Net network, which enhances the nodal feature information and improves the segmentation performance of lung nodules. Although the 2D segmentation network achieves better segmentation results in some cases, it still suffers from information loss and is susceptible to local disturbances that produce erroneous segmentation results when dealing with the 3D structure of lung nodules. Among the 3D network segmentation models, Chen et al.^23^ proposed an improved segmentation model, VBNet, based on V‐Net and MobileNet‐V2, which can obtain a larger receptive field and reduce the occurrence of mis‐segmentation without increasing the network depth. Zhong et al.^24^ introduced a multi‐scale feature structure based on the VNet architecture, so that the whole network is integrated by four VNet sub‐networks with different scales to adaptively select the optimal segmentation path. Although the 3D network can well utilize the 3D structural information of lung nodules, it has high computational complexity, large memory consumption, and high requirements in terms of training sample requirements and data quality. For 3D objects with isotropic resolution, the 2.5D network segmentation model achieves a good trade‐off between model complexity, sensory domain, and GPU memory and achieves competitive performance with respect to the 3D segmentation model and the 2D model.^25^ Chen et al.^26^ proposed inputting 2D and 3D images into a branching network, which is used to capture contextual feature information from multidimensional adjacent axes. The contextual features are then fused using a global convolutional layer. Ni et al.^27^ proposed a multi‐task 2.5D MSU‐Net structure that employs separable convolution to preserve the hierarchical features of the 2.5‐dimensional structure, and a multi‐scale input layer to achieve the fusion of small‐scale features with large‐scale features. In the lung nodule segmentation task, although 3D segmentation models generally yield poorer DSC performance than 2D segmentation models,^16^ since CT images of lung nodules contain 3D information, a network model with a three‐dimensional structure better prevents mis‐segmentation from occurring, and 2.5D networks combining both 2D and 3D convolutions have been shown to be more efficient than standard 3D networks.^28^ Although the segmentation of lung nodules by 2.5D networks has achieved certain results, the existing methods still have limitations in dealing with the complexity of lung anatomical structures and high noise interference. Therefore, to address these challenges, our study aims to further improve and optimize the existing segmentation methods for lung nodules in 2.5D networks in order to increase the accuracy, robustness, and efficiency of segmentation.

In this study, we propose a 2.5D‐based V‐Net network framework. The network replaces the original 3D convolution with a separable 3D (S3D) convolution^29^ to enable the network to have both inter‐slice and intra‐slice features, and incorporate a dual‐branch feature fusion module (DFFM)^30^ and an reverse attention context module (RACM)^30^ to enhance the lung nodule features and edge features for more accurate edge segmentation results. In addition, we use a central pooling layer instead of the uniformly distributed maximum pooling layer of the original network, which is used to retain more information about the desired target and improve the feature extraction performance.

## 2 MATERIALS AND METHODS

### 2.1 Network structure in this paper

**FIGURE 2:**
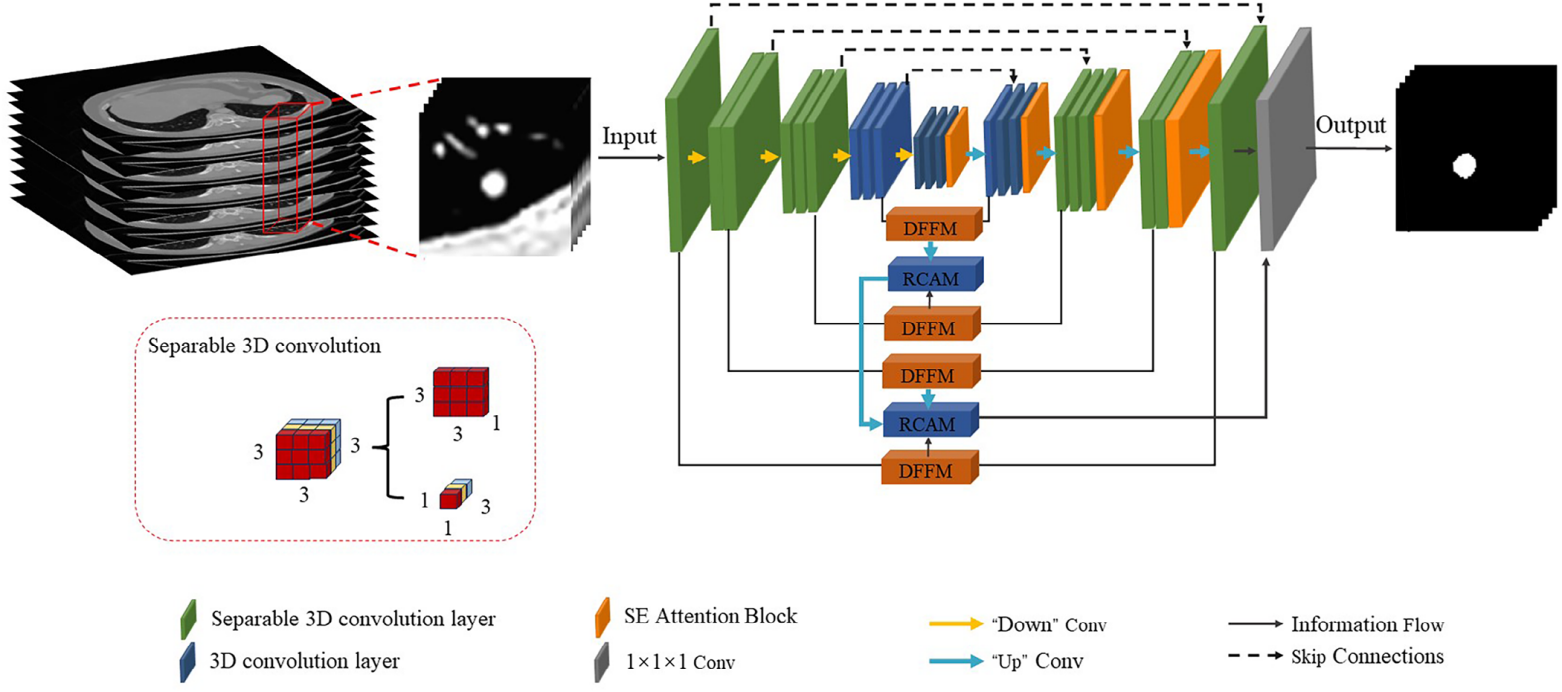
Proposed 2.5D V‐Net lung nodule segmentation network.

Our proposed 2.5D V‐Net network model is shown in Figure 2, and the main structure follows the widely used V‐Net network,^18^ where the residual module in V‐Net can well solve the gradient vanishing/inflating problem in neural networks. In this paper, the network uses a series of region of interest (ROI) slices with an 11‐layer image structure as inputs to segment the lung nodules using 3D spatial information and 2D planar information, which takes advantage of the 2D network, and the use of a separable 3D convolution also avoids the damage caused by the 2D convolution kernel to the structure. Moreover, the use of a DFFM to fuse the feature mappings of the same layer and through the RACM used to provide multilevel semantic information fully achieves the information complementarity between features, so that lung nodules with fuzzy edges can be accurately segmented.

### 2.2 Separable 3D convolution layer

Since the variance of the weight distributions between the slices is larger the lower the level, and the variance of the distributions of different weights within the slices is larger the top the level,^29^ this suggests that the 3D convolution operation is more meaningful the top the level, and the replacement of the 3D convolution by the S3D convolution the lower the level has a lower impact on the network, and the amount of computation is reduced dramatically. Therefore, we use S3D convolution to replace the original 3D convolution at the last two levels of the network (L4‐L5). The S3D structure is shown in the blue dotted box in Figure 2, where a 1 × 3 × 3 convolution kernel is used to perform 2D intra‐slice convolution, and then a 3 × 1 × 1 convolution kernel is used to perform 1D inter‐slice convolution to replace the original 3 × 3 × 3 convolution kernel. This network has a smaller number of parameters, higher computational efficiency, and better accuracy than the network using standard 3D convolution. And after each convolution, we perform batch normalization (BN)^31^ to ensure the stability and convergence of the training.

### 2.3 DFFM and RACM

The encoder part of the V‐Net network generates low‐level semantically informative features and high‐resolution features in the low‐level network, and high‐level semantically informative features and low‐resolution features in the high‐level network; feature fusion is performed in the decoder part to predict the segmentation results. However, the V‐Net network^18^ is rather limited in its skip connections, and feature fusion can only be performed on the same proportion of feature maps in the encoder and decoder subnetworks. In this process, the network only focuses on complex features at a single scale, ignoring the contribution of features at other scales to the lung nodule detection task, and thus this approach achieves little result.^32^

**FIGURE 3:**
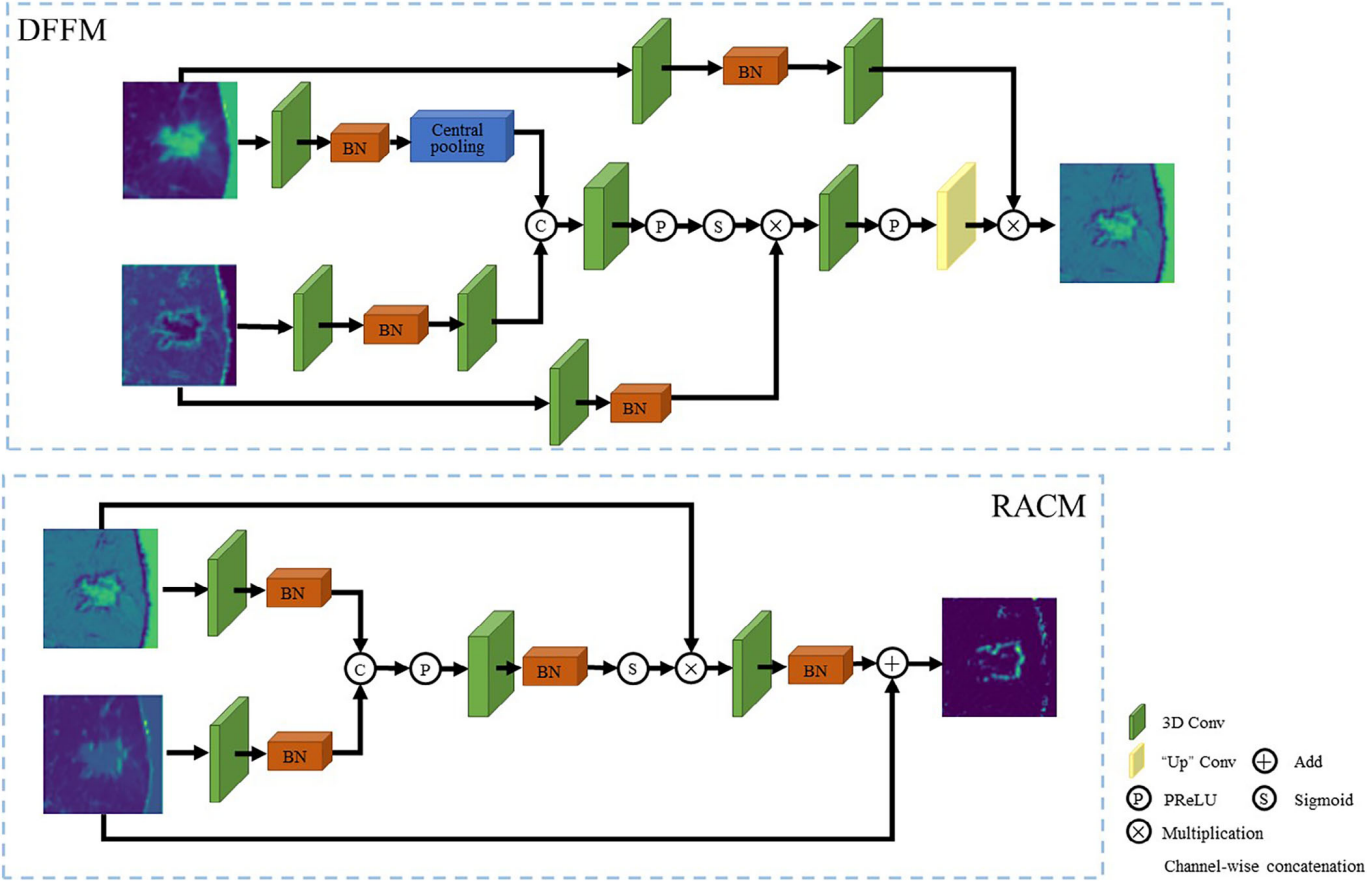
The dual‐branch feature fusion module and the reverse attention context module structure.

Therefore, we construct another DFFM between encoding and decoding. Although it also only fuses the feature shadows of the same layer, this module is more adaptive to integrate local features and global dependencies, leaving more effective information compared to the original network. The structure of our DFFM is shown in Figure 3. Experiments with our dataset yielded results that we visualize in images, which we show as sliced features, but which are actually 3D data. In this structure, two feature maps of the same size obtained from the encoder and decoder are resolution adjusted by a 3 × 3 × 3 convolutional kernel, and then the features obtained from the encoder are maximally pooled to obtain the region of most interest. This process prevents the loss of small targets during training and outputs better feature pooling results.

In lung nodule image segmentation, accurate segmentation of edge information enables doctors to make better diagnosis of lung nodules. Therefore, we use a RACM and insert it into the feature fusion module to enhance edge features and improve segmentation accuracy. The details of RACM are shown in Figure 3. In this process, we connect the two RACMs in series and add jump connections so that they can make full use of deeper spatial features and obtain more accurate edge segmentation results. Finally, we input the result to the original decoder to enhance the detailed features. In addition, we also introduce the Squeeze‐Excite (SE) block^33^ in the original decoding path to assign different weights to the channel features in order to improve the performance of the network.

### 2.4 3D central pooling

The input data for the network in this paper is a series of slices central on lung nodules, therefore, we use central pooling^13^ in the segmentation instead of the maximum pooling, which is used to preserve the features around the target and eliminate the edge irrelevant features. We use 2D central pooling and 1D central pooling to implement the 3D central pooling function. The size of the pooling kernel when we use 2D central pooling varies with the location of the pooling, in the central we use a small pooling kernel (s = 1) and at the edges we use a large pooling kernel (s = 3), and to ensure that the size after passing through this pooling is the same as the size after the previous pooling we also set half the number of the pooling kernels to the pooling kernel of (s = 2).

Given a volume block of size Q × Q × Z, and n1, n2, and n3 represent three pooling kernels of different sizes, and their quantitative relationship can be given by the following equation:

(1) $$\begin{array}{c} \left\{\begin{array}{c} n1+2n2+3n3\;=\;Q\\ n1+n2+n3\;=\frac{Q}{2}\;\;\\ n1+n3\;=\;n2\; \end{array}\right. \end{array}$$

**FIGURE 4:**
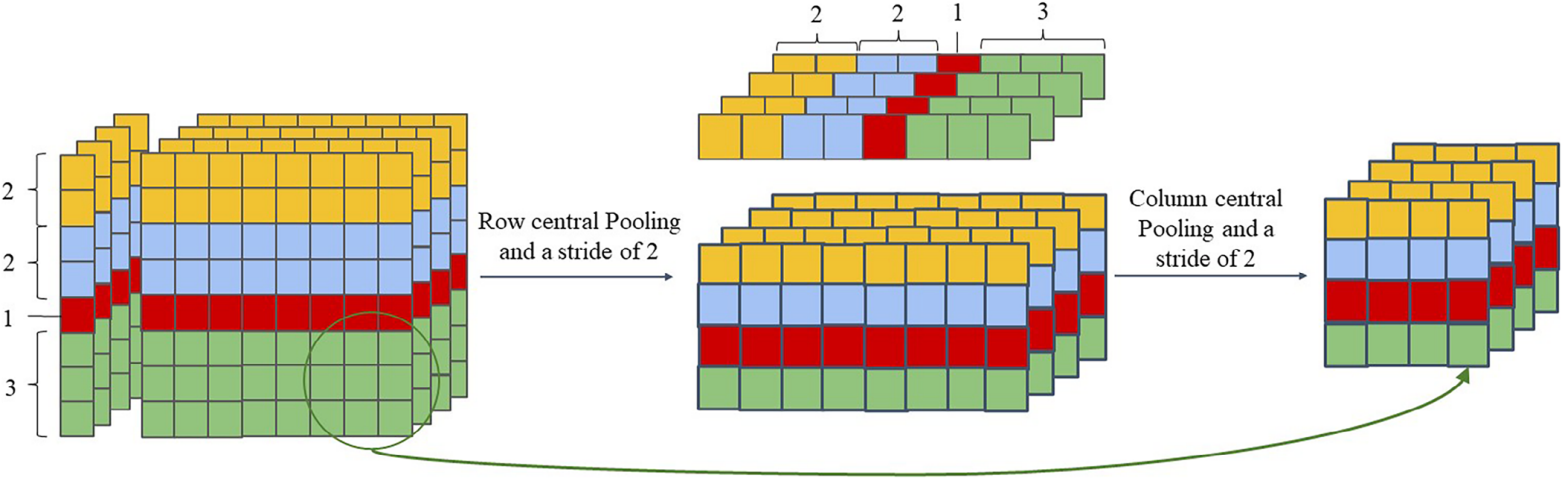
Central pooling process.

According to the size of our input data, we can get the number of n1 is 8, the number of n2 is 16, and the number of n3 is 8. We assign the set kernels of different sizes to the set of input slices in a centrally symmetric way, and take the four inputs of 8 × 8 as an example, we can get n1 = 1, n2 = 2, and n3 = 1 by Equation (3), and the central set process is shown in Figure 4.

### 2.5 Datasets and data preprocessing

In order to evaluate the performance and efficiency of the proposed segmentation method, we used a dataset from the Lung Nodule Analysis 2016 (LUNA16) competition, which was launched by Colin et al.^34^ The dataset contains 1186 nodules accepted by at least three of four radiologists divided into a total of 10 subsets, so this study uses a rule of 10 cross‐validation. Nodule diameters in this dataset range from 3.170  to 27.442 mm, and the slice interval ranges from 0.45 to 3 mm, the axial plan resolution varies from 0.46 mm × 0.46 mm to 0.98 mm × 0.98 mm.

**FIGURE 5:**
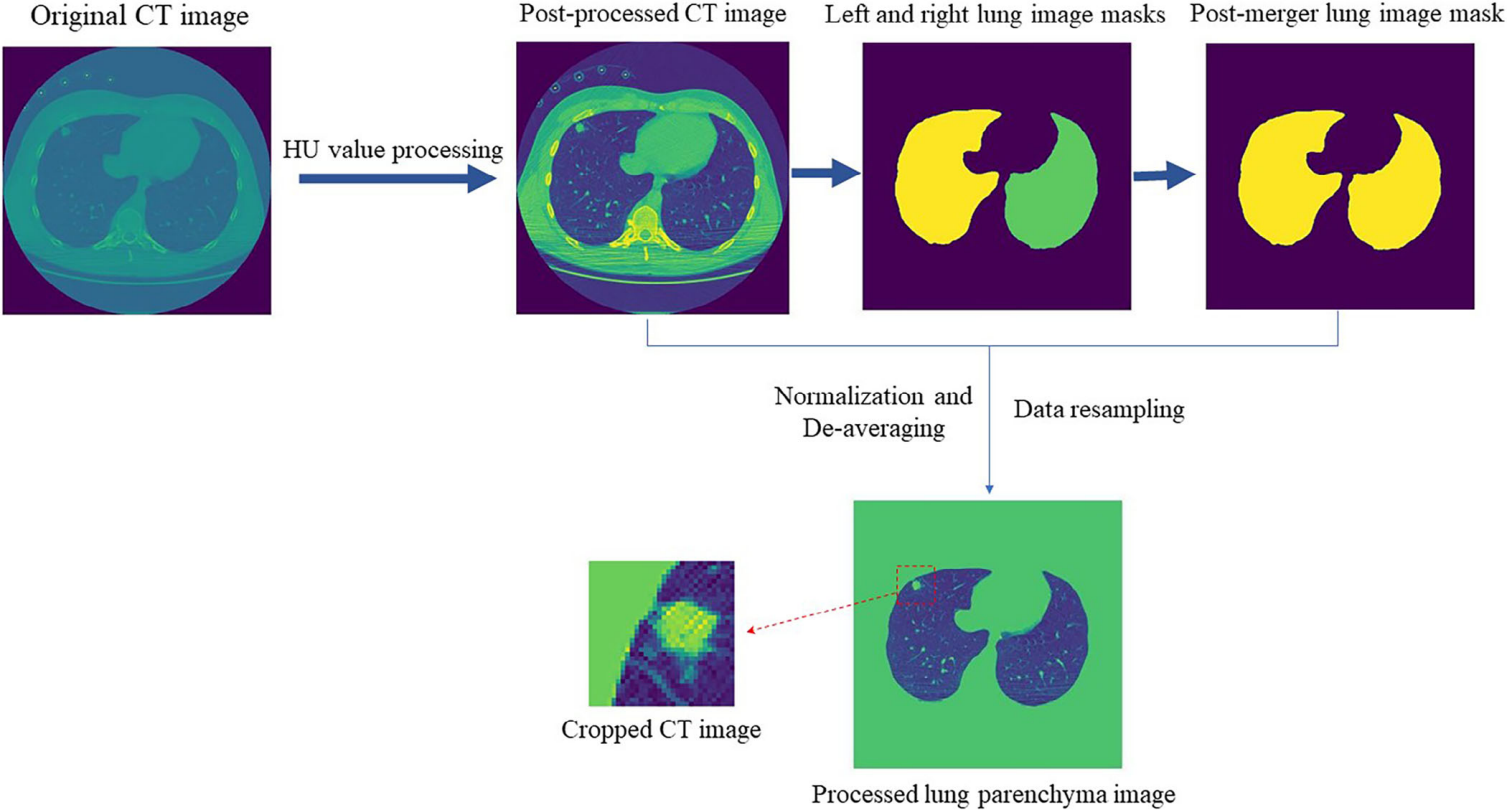
Data preprocessing. The data were preprocessed and the size of each slice after processing was 64 × 64.

In the data preprocessing stage, we converted the original CT images into HU values and retained the range of HU values [−1000,600],^24^ which was used to reduce the influence of other regions such as air and water in the lung CT on the segmentation of lung nodules, and then extracted the lung mask to obtain the CT images of the lung region only. After that, the data were resampled to ensure that the pixel spacing in the x, y, and z directions of the images were all 1 mm, and the raw data were normalized and de‐averaged to eliminate the errors caused by different sampling intervals. Finally, centered on the lung nodules labeled by the doctor, the slices were cropped to 64 × 64 size and trained as a set of slices. The data preprocessing is shown in Figure 5.

### 2.6 Experiment and evaluation metrics

The experiments in this paper use ten rules of cross‐validation,^35^ where the dataset is divided into 10 parts, rotate nine of them as training data and one as test data, and the average of the 10 results is used as an estimate of the algorithm's accuracy. For the parameter settings, the initial learning rate was set to 3e−4,^36^ the batch size was set to 8, and the maximum number of training iterations was set to 200. We use a dynamic learning rate to prevent overfitting, where the learning rate decreases by 0.01 for every 30 epochs of training. During training, we generate far fewer parameters than most other 3D networks, with faster processing power. And the models used in this paper were all performed in Python 3.8.15 based on the PyTorch 1.13.1^37^ deep learning framework, and the graphics processor used (GPU) was an NVIDIA GeForce RTX 3060.

For the evaluation metrics, we use DSC and overlapping error (OE) to be the evaluation indexes for pulmonary nodule segmentation.^38^ The DSC is often used as a measure of segmentation, we use it to measure the ratio of the pixels of the set of standard and predicted segmentation pixels to the sum of their total pixels. The OE is calculated based on the 1‐Jaccard Index, and the Jaccard Index is calculated by calculating the ratio of the pixels intersecting the standard and predicted segmentation pixels to the pixels in their concatenation.

DSC is calculated as follows:

(2) $$\begin{array}{c} \text{DSC}=\frac{2\left|A\cap B\right|}{\left|A\right|+\left|B\right|}\;=\frac{2\text{TP}}{2\text{TP}+\text{FP}+\text{FN}} \end{array}$$

OE is calculated as follows:

(3) $$\text{OE}=\;1-\;\frac{\left|A\cap B\right|}{\left|A\cup B\right|}=\;1-\frac{\text{TP}}{\text{TP}+\text{FP}+\text{FN}}$$

where A and B are the standard segmentation results and predicted segmentation results, and TP, FP, and FN represent the true positive, false positive, and false negative, respectively.

For loss function, Dice loss^18^ is widely used in medical image segmentation, the Dice Similarity Coefficient (DSC) is usually used to calculate the similarity of two samples. Dice loss is calculated as follows:

(4) $$\begin{array}{c} \;L_{dice}=\;1-\text{DSC}=\;1-\frac{2\left|A\cap B\right|}{\left|A\right|+\left|B\right|} \end{array}$$

where A and B are the standard segmentation results and predicted segmentation results.

In this experiment, the input data are cropped CT images, which alleviate the pixel imbalance problem in foreground pixels and background pixels, so we choose a hybrid loss function of Binary Cross Entropy (BCE) and Dice loss as the loss function of the network in this paper. The BCE loss function as follows:

(5) $$\begin{array}{c} \;L_{bce}=\;-w_{p}\left[y_{p}\log x_{p}+\left(1-y_{p}\right)\log\left(1-x_{p}\right)\right] \end{array}$$

where $w_{p}$ represents the weight for balancing positive and negative samples, $y_{p}$ represents the target sample, and $x_{p}$ represents the predicted sample.

The BCEDice loss function in this paper is as follows:

(6) $$\begin{array}{c} \;L_{bcedice}=\;aL_{dice}+\left(1-a\right)L_{bce} \end{array}$$

where a represents the weight for $L_{dice}$ and $L_{bce}$. In the experiment, we conducted one training at a value of 0.3, 0.5, and 0.7, respectively; the results are shown in Table 1 and found that the loss value is more able to reach the minimum when the value of a is 0.5, so the final training in this paper chooses the value of a to be 0.5.

**TABLE 1:** Different weighting results.

Loss function	a	mDSC (%)	mOE (%)
Dice	–	83.44	21.14
BCE	–	82.59	20.93
	0.3	83.62	20.31
BCEDice	0.5	84.57	18.73
	0.7	84.19	19.16

## 3 RESULTS

In order to verify the validity of the methodology of this study, in Table 2, we compare the methodology of this paper with the more commonly used 2D, 3D, and 2.5D methods. For a fair comparison, we used the same database and experimented with its officially released code, by comparing our method obtained the highest mDSC vaule of 84.57%, and mOE value also obtained the lowest score of 18.73%, and average processing of a case is about 2.07 s, also performs well compared to other common 2.5D split networks. Our results were compared with the inter‐radiologist consistency on the LUNA16 dataset, with an average difference of only 0.74%. This means that among these networks, our segmentation network is more effective for the lung nodule segmentation task. Comparing the amount of computation and number of parameters required by different methods, we also have a better performance. In addition, our network performs better than the MSU‐Net network and the DFF‐Net network, which suggests that the two‐branch feature fusion module as well as the improved module in our decoding are more effective than multi‐scale fusion and single two‐branch feature fusion. After performing ten cross validations for each model, we use *t*‐test to compare whether there is a significant difference in the performance of DSC values derived from different models and our model in ten cross validations. The results are shown in Table 3, and by comparing the *p*‐values, we can conclude that there is a significant difference between our model and the previous model.

**TABLE 2:** Comparison results of different segmentation methods.

Model	Dim	mDSC (%)	mOE (%)	Parameter (M)	Calculation (GMAC)	Time (s)
U‐Net^17^	2D	74.17	43.77	31.04	3.43	1.64
U‐Net++^36^	2D	80.83	27.64	26.07	1.15	1.83
SGU‐Net^39^	2D	82.73	20.16	4.99	0.31	1.15
DFF‐Net^30^	2D	83.87	19.13	13.56	2.73	1.74
U‐Net^40^	3D	79.52	31.15	16.32	237.01	2.73
V‐Net^18^	3D	77.81	30.56	45.60	93.91	2.31
U‐Net++^36^	3D	81.23	25.13	5.53	49.2	1.98
U‐Net^41^	2.5D	80.65	32.43	20.46	83.14	2.14
MSU‐Net^27^	2.5D	83.25	21.57	24.36	95.28	2.35
**Our model**	**2.5D**	**84.57**	**18.73**	**38.28**	**52.64**	**2.07**

Abbreviations: Dim, Dimension; DSC, Dice Similarity Coefficient; OE, overlapping error.

**TABLE 3:** Results of *t*‐test analysis.

Model	mDSC(%) ± SD	*t*	*p*
U‐Net^17^	74.17 ± 1.69	16.74	<0.001
U‐Net++^36^	80.83 ± 1.03	8.22	<0.001
SGU‐Net^39^	82.73 ± 0.73	4.66	<0.001
DFF‐Net^30^	83.87 ± 0.93	1.61	0.124
U‐Net^40^	79.52 ± 1.47	8.96	<0.001
V‐Net^18^	77.81 ± 1.59	11.38	<0.001
U‐Net++^36^	81.23 ± 0.95	7.61	<0.001
U‐Net^41^	80.65 ± 0.99	8.77	<0.001
MSU‐Net^27^	83.25 ± 0.68	3.45	0.001

Abbreviation: SD, standard deviation.

**FIGURE 6:**
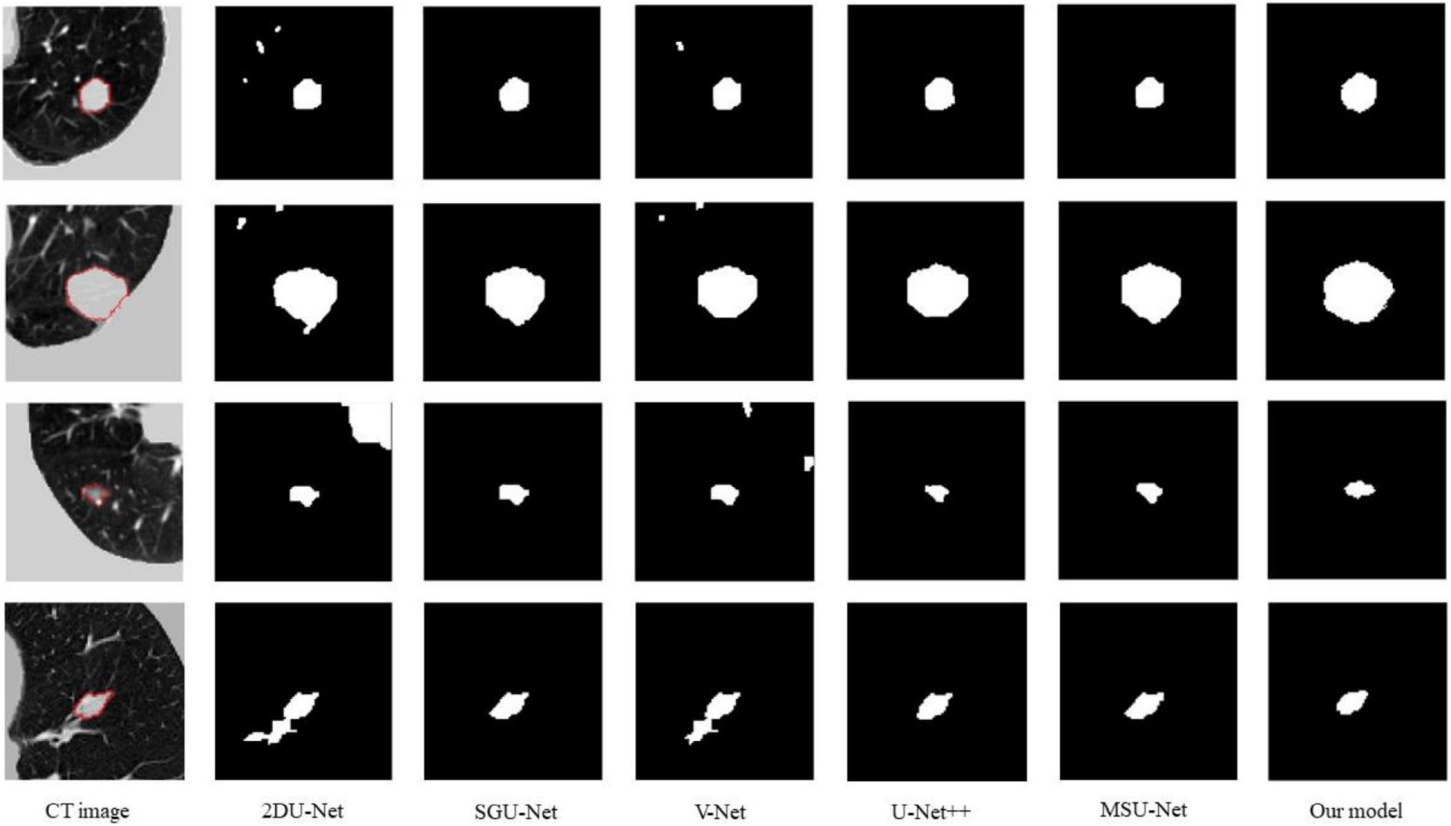
Comparison of segmentation results of different segmentation methods.

We give a visual plot of the segmentation comparison in Figure 6, where column 1 shows the consensus segmentation of the preprocessed CT images. Counting from top to bottom the first case is a solid isolated nodule, the second case is a lung wall adherent nodule, the third case is a GGO nodule, and the fourth case is a vascular adherent nodule. Our network model can reduce the mis‐segmentation phenomenon of the lung nodule very well compared with the 2D‐Unet and V‐Net network models, and retains good detailed features, and the segmentation result of our nodule is closest to the standard segmentation compared with other networks, which suggests that our method can obtain better segmentation results.

## 4 DISCUSSION

In addition to comparisons with other methods, in order to confirm that each of the modules we have added has a positive effect, we have also carried out an ablation study for each of the components in the model, which is discussed in detail. In Table 4, we give some intuitive results of the removal of each component. It can be seen that the segmentation metrics decrease when we remove DFFM, RACM, and other modules from the network, respectively, while the best evaluation metrics can be obtained when these modules act simultaneously, which indicates that the modules we use are reasonable and effective.

**TABLE 4:** Results of ablation experiments.

Model	mDSC (%)	mOE (%)	Parameter (M)	Calculation (GMAC)
①V‐Net^18^	77.81	30.56	45.60	93.91
②S3DV‐Net	78.32	30.34	33.47	47.26
③S3DV‐Net+DFFM	81.71	21.85	35.67	49.54
④S3DV‐Net+DFFM+RACM	82.25	20.57	37.61	51.78
⑤S3DV‐Net+DFFM+RACM+C	83.87	19.22	37.64	51.83
⑥S3DV‐Net+DFFM+RACM+C+SE	84.57	18.73	38.28	52.64

**FIGURE 7:**
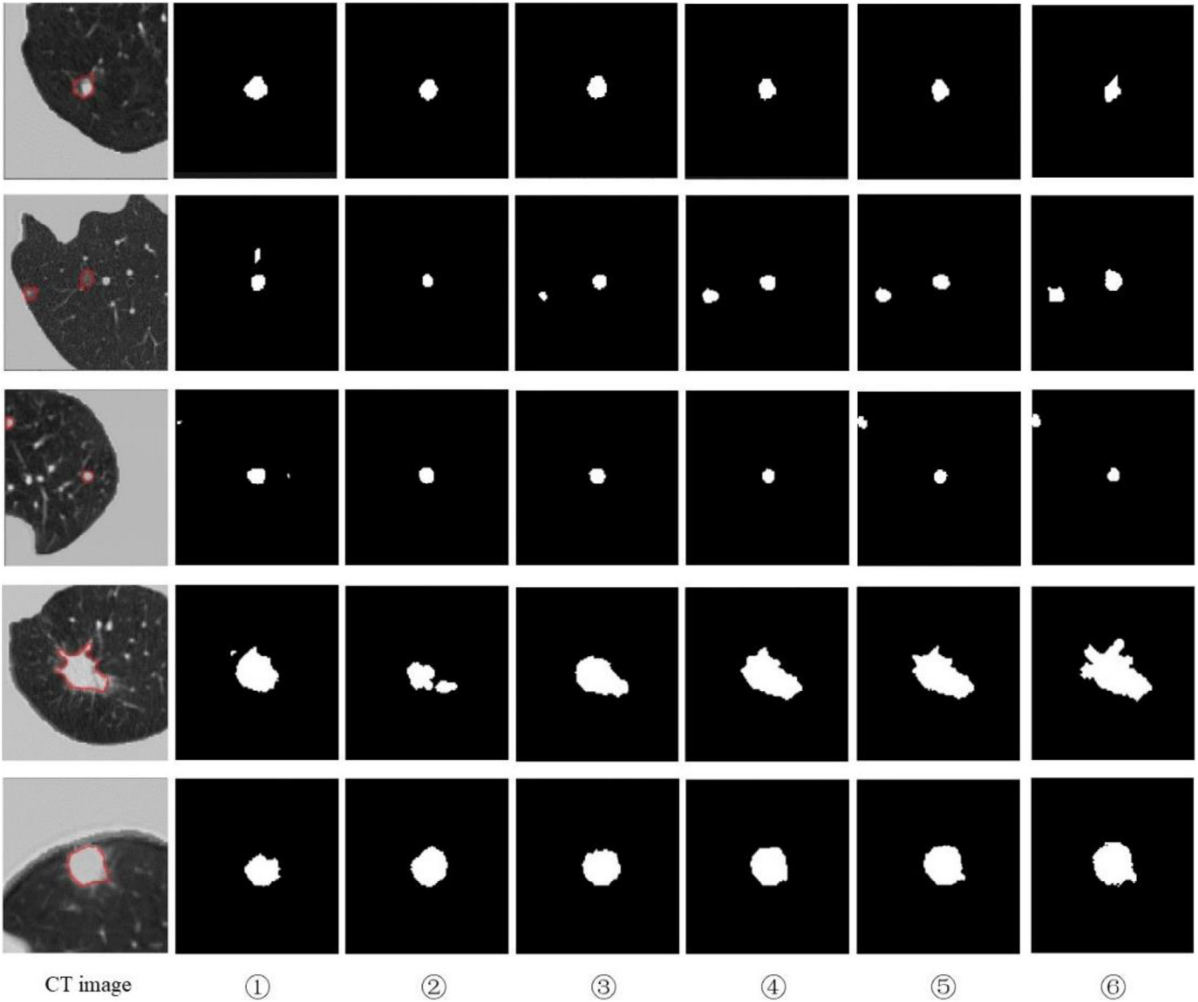
Intuitive results for each component. ①②③④⑤⑥represent the individual module networks in Table 4.

Figure 7 shows the comparison of intuitive segmentation results after removing each module, and we can see that this paper has better performance after adding each module, and the final network model segmentation is the best. We can observe that after removing the DFFM, the segmentation accuracy is poor and there is false positive segmentation; after removing the RACM, the segmented lung nodule edge features are not obvious; meanwhile, after adding the SE module and improving the pooling method, the edge features of the nodules are more prominent and the segmentation accuracy is higher. Therefore, the dual‐branch feature fusion module and the improved central pooling network model in this paper can improve the extraction of pulmonary nodule edge features, effectively reduce the phenomenon of mis‐segmentation of pulmonary nodules, and have better segmentation effect on the edge of pulmonary nodules.

DDFM, RACM, and central pooling, which can fuse multi‐resolution feature maps to guide the accurate segmentation of small targets with richer feature information, and RACM can enhance the edge texture of features to help feature recovery and mitigate the loss of accuracy caused by edge blurring. Center pooling has good performance in the input data centered on lung nodules in this paper, highlighting the feature information of lung nodules. And the comparison experiments on the LUNA16 dataset prove the superiority and robustness of our network. The ablation experiments further validate the rationality and practicality of our improved module. In conclusion, this research network provides an effective solution for lung nodule segmentation. In the future, we will further explore 3D data enhancement techniques to increase the diversity of data thereby improving the performance of the segmentation model, and extend the proposed method to other segmentation tasks.

## 5 CONCLUSION

In this study, we propose a new two‐branch fusion attention model for lung nodule segmentation based on the V‐Net network, and the results show that our method has a better improvement for lung nodule segmentation accuracy, which can well fuse the multi‐resolution feature maps, enhance the edge texture of the features, and mitigate the loss of accuracy caused by edge blurring, which provides an effective solution and potential value for lung nodule segmentation.

## AUTHOR CONTRIBUTIONS

**Xiaoru Xu**: Conceptualization; methodology; writing—original draft preparation; writing—review and editing. **Lingyan Du**: Conceptualization; writing—review and editing. **Dongsheng Yin**: Methodology; writing—review and editing. All authors have read and agreed to the published version of the manuscript.

## CONFLICT OF INTEREST STATEMENT

The authors declare no conflict of interest. If there is a need for the code, you can contact the authors, after evaluation can be given.

## Data Availability

The data that support the findings of this study are openly available in the Lung Image Database Consortium (LIDC) and Image Database Resource Initiative (IDRI): A completed reference database of lung nodules on CT scans (LIDC‐IDRI) at https://arxiv.org/abs/1612.08012, reference number [34].
